# Inner Workings of Arsenic: DNA Methylation Targets Offer Clues to Mechanisms of Toxicity

**DOI:** 10.1289/ehp.123-A21

**Published:** 2015-01-01

**Authors:** Lindsey Konkel

**Affiliations:** Lindsey Konkel is a Worcester, MA–based journalist who reports on science, health, and the environment.

Arsenic is a known human carcinogen,^1^ although it’s unclear how it causes cancer. Some studies have suggested that epigenetic modifications—specifically DNA methylation—may play a role in arsenic toxicity.^2^ In this issue of *EHP*, investigators identify gene-specific DNA methylation targets in white blood cells in a large study of Bangladeshi adults.^3^

In addition to cancer, chronic exposure to arsenic in drinking water has been associated with an increased risk of cardiovascular disease, peripheral neuropathy, respiratory diseases, and diabetes.^4^ Previous epidemiological studies probing DNA methylation and arsenic exposure have isolated methylation patterns within specific genes of interest,^5^^,^^6^ and a few have begun to assess epigenome-wide changes.^7^^,^^8^^,^^9^ The new study is the largest to date to investigate arsenic-related changes throughout the epigenome.

**Figure:**
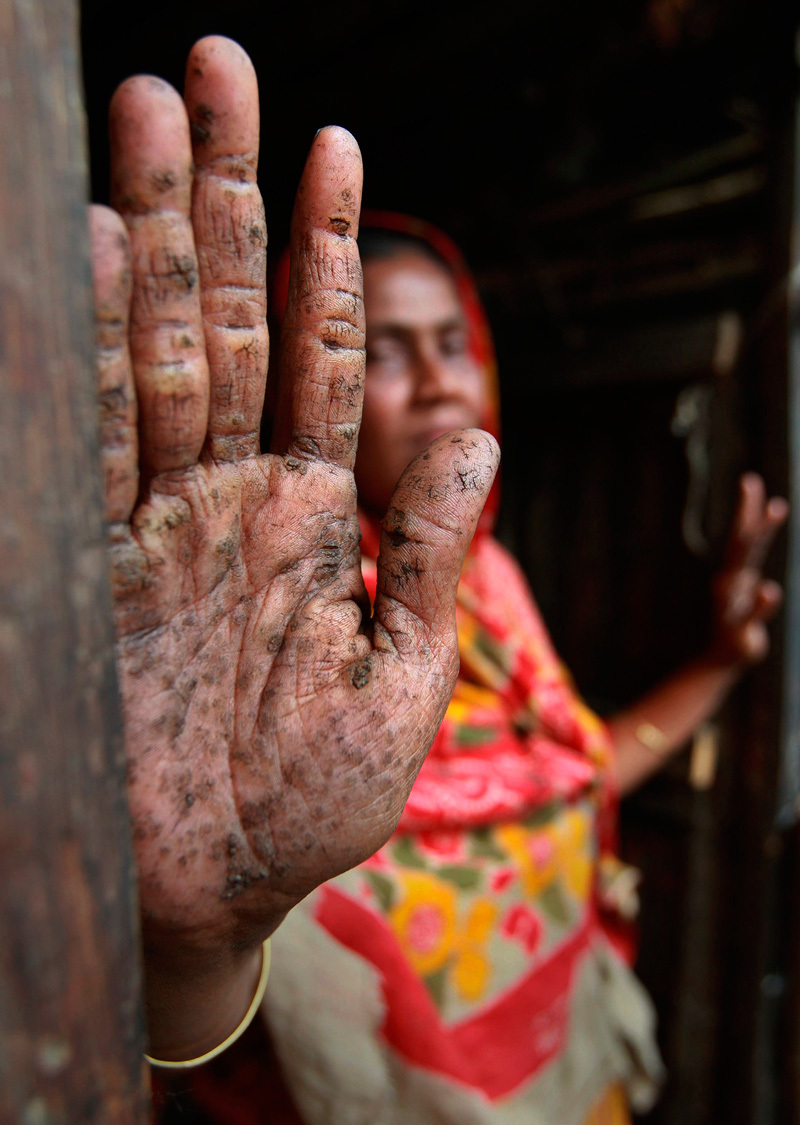
Skin lesions can occur with any level of arsenic exposure but are most prevalent among people of South Asian descent, suggesting a genetic component. © AP Photo/Pavel Rahman

“Many of the genes we identified happened to be related to pathways that are associated with skin cancer, which was very relevant to the study population,” says lead author Maria Argos, an epidemiologist at the University of Illinois at Chicago. The researchers used blood and urine samples provided by more than 400 adults from rural Bangladesh with arsenical skin lesions—thickened or blackened areas of the skin that are associated with chronic arsenic exposure. People with arsenical skin lesions may be at an increased risk for developing skin cancer.^10^

Four gene loci showed significant changes in methylation status in relation to urinary arsenic concentration. Three of these also showed significant changes in relation to blood arsenic concentration. These four loci—sites in the genes *PLA2G2C*, *SQSTM1*, *SLC4A4*, and *IGH*—had not previously been associated with arsenic exposure.^3^

The researchers observed that several of the differentially methylated loci were associated with changes in gene expression levels in white blood cells.^3^ “The changes in gene expression that we observed make sense in the arsenic pathology that we see. These are good clues for exploring mechanism and prevention avenues,” says senior study author Habibul Ahsan, a medical epidemiologist at the University of Chicago.

For instance, *PLA2G2C* encodes lipid mediators with roles in inflammation, cell growth, and cell death,^11^ making them potentially important for cancer progression.^3^ Higher arsenic exposure was associated with decreased methylation levels at a locus of the *SQSTM1* gene that has been implicated in a number of diseases, including cancer, obesity, insulin resistance, and neurodegenerative diseases.^12^

“The addition of gene expression data to DNA methylation data makes this study very unique and begins to suggest how some of the epigenetic changes may be linked to downstream health outcomes,” says Carmen Marsit, a molecular epidemiologist at the Geisel School of Medicine, Dartmouth College, who was not involved with the study.

The researchers identified methylation patterns in blood cells, but every tissue type in the body has a different methylation pattern. “We can’t necessarily say that the DNA methylation profile associated with arsenic exposure in blood would be the same as in skin or other tissues of the body,” Argos says.

The majority of study participants had higher levels of arsenic exposure than would typically be seen in the U.S. population, says Ahsan. And although skin lesions can develop at any level of arsenic exposure, he says, they are more widespread among people of South Asian descent, suggesting a genetic component. “It’s not clear yet how important a factor genetic variation may be in setting DNA methylation patterns,” Marsit says.

According to Argos, epidemiological studies may be able to use DNA methylation patterns in blood as a surrogate for past exposures to arsenic. Scientific research combining DNA methylation and gene expression data is a step toward doing more integrative molecular studies, she says. “We showed there is a lot to be learned by overlaying different types of molecular data.”

## References

[1] IARC. Arsenic, Metals, Fibres, and Dusts. IARC Monographs on the Evaluation of Carcinogenic Risks to Humans. Volume 100 C: A Review of Human Carcinogens. Lyon, France:International Agency for Research on Cancer (2012). Available: http://monographs.iarc.fr/ENG/Monographs/vol100C/mono100C.pdf [accessed 5 December 2014]

[2] Reichard JF Puga A Effects of arsenic exposure on DNA methylation and epigenetic gene regulation. Epigenomics 2 1 87 104 2010; 10.2217/epi.09.45 20514360 PMC2877392

[3] Argos M Gene-specific differential DNA methylation and chronic arsenic exposure in an epigenome-wide association study of adults in Bangladesh. Environ Health Perspect 123 1 64 71 2015; 10.1289/ehp.1307884 25325195 PMC4286273

[4] Navas-Acien A Arsenic exposure and type 2 diabetes: a systematic review of the experimental and epidemiologic evidence. Environ Health Perspect 114 5 641 648 2006; 10.1289/ehp.8551 16675414 PMC1459913

[5] Engström KS Efficient arsenic metabolism—the *AS3MT* haplotype is associated with DNA methylation and expression of multiple genes around *AS3MT*. PLoS ONE 8 1 e53732 2013; 10.1371/journal.pone.0053732 23341986 PMC3544896

[6] Intarasunanont P Effects of arsenic exposure on DNA methylation in cord blood samples from newborn babies and in a human lymphoblast cell line. Environ Health 11 1 31 2012; 10.1186/1476-069X-11-31 22551203 PMC3506565

[7] Smeester L Epigenetic changes in individuals with arsenicosis. Chem Res Toxicol 24 2 165 167 2011; 10.1021/tx1004419 21291286 PMC3042796

[8] Bailey KA Arsenic and the epigenome: interindividual differences in arsenic metabolism related to distinct patterns of DNA methylation. J Biochem Mol Toxicol 27 2 106 115 2013; 10.1002/jbt.21462 23315758 PMC3892431

[9] Koestler DC Differential DNA methylation in umbilical cord blood of infants exposed to low levels of arsenic *in utero*. Environ Health Perspect 121 8 971 977 2013; 10.1289/ehp.1205925 23757598 PMC3733676

[10] Rahman M Prevalence of arsenic exposure and skin lesions. A population based survey in Matlab, Bangladesh. J Epidemiol Community Health 60 3 242 248 2006; 10.1136/jech.2005.040212 16476755 PMC2465558

[11] Dennis EA Phospholipidase a2 enzymes: physical structure, biological function, disease implication, chemical inhibition, and therapeutic intervention. Chem Rev 111 10 6130 6185 2011; 10.1021/cr200085w 21910409 PMC3196595

[12] Geetha T Sequestosome 1/p62: across diseases. Biomarkers 17 2 99 103 2012; 10.3109/1354750X.2011.653986 22296116

